# Neural responses to action contingency error in different cortical areas are attributable to forward prediction or sensory processing

**DOI:** 10.1038/s41598-019-46350-1

**Published:** 2019-07-08

**Authors:** Tatsuo Kikuchi, Motoaki Sugiura, Yuki Yamamoto, Yukako Sasaki, Sugiko Hanawa, Atsushi Sakuma, Kazunori Matsumoto, Hiroo Matsuoka, Ryuta Kawashima

**Affiliations:** 1 Institute of Development, Aging and Cancer, Tohoku University, Seiryo-machi 4-1, Aoba-ku, Sendai 980-8575 Japan; 2 Department of Psychiatry, Tohoku University Graduate School of Medicine, 2-1 Seiryo-machi, Aoba-ku, Sendai, 980-8574 Japan; 3 International Research Institute of Disaster Science, Tohoku University, Aramaki-aza-aoba 468-1, Aoba-ku, Sendai 980-0845 Japan; 4 Tohoku University Hospital, 1-1 Seiryo-machi, Aoba-ku, Sendai, 980-8574 Japan

**Keywords:** Perception, Motor cortex, Disorders of consciousness

## Abstract

The contingency of sensory feedback to one’s actions is essential for the sense of agency, and experimental violation of this contingency is a standard paradigm in the neuroscience of self-awareness and schizophrenia. However, neural responses to this violation have arbitrarily been interpreted either as activation of the system generating forward prediction (agency-error account) or decreased suppression of processing of predictable input (prediction-error account). In this functional magnetic resonance imaging (fMRI) study, the regions responsive to auditory contingency errors were examined if they exhibited responses to an isolated auditory stimulus and to passive-contingency delay, which the prediction-error account expects. These responses were observed only in the auditory association cortex in the right superior temporal gyrus. Several multimodal and motor-association cortices did not exhibit these responses, suggesting their relevance to the agency-error account. Thus, we formulated the coexistence and dissociation of two accounts in neural contingency-error responses.

## Introduction

The sense of agency refers to the feeling of one’s own movement being caused or controlled by oneself, and it forms the essential basis of self-consciousness^1,2^. Disturbance of the self is the core pathology of schizophrenia and dysfunction of the mechanism underlying the sense of agency is considered responsible for this pathology^3,4^. Therefore, cognitive neuroscience research on the sense of agency has both scientific and clinical significance.

In the influential framework of the forward model of motor control, the sense of agency is primarily determined by the contingency of sensory feedback to one’s actions^2,5,6^. When an action is executed, the motor commands are sent to the motor system^7^. In the framework, we implicitly predict the sensory feedback that would result from the action, based on the motor commands and internal model of the body and the physical environment^8,9^. This forward prediction of consequential sensory feedback is compared with actual sensory input for the online monitoring and adjustment of the motor action to allow efficient control^10,11^. The comparison is implemented as the subtraction of predicted input from actual input in sensory processing, thus leaving the differential component for higher-level processes^7^. Sensory processing is mostly suppressed in an ideal action situation where sensory feedback is perfectly contingent on the action; functioning is felt as the sense of agency or unconscious^7,12^. When there is some mismatch between the predicted and actual inputs due to improper functioning of motor control system or external forces, the differential component in sensory processing triggers the compensation for action deviation, recalibration of the internal model, or the violated sense of agency and increased self-monitoring^8,9,13–16^.

Therefore, in neuroimaging studies, the neural response to contingency error is recognized as the index of neural correlates of the sense of agency. An agent learns the contingent relationship between an action and its sensory feedback through repeated experiences, and the acquired association enables an agent to feel the sense of agency^2,7,14,17^. Experimental manipulation of contingency reduces the sense of agency^3^ and activates various brain regions. This manipulation usually consists of temporal delay^3,18–22^ or spatial violation^23–27^. For example, in a previous study, feedback tone was immediately delivered when subjects pressed a button in the “predictable” condition but was randomly delayed in the “unpredictable” condition^18^. Another study accustomed subjects to moving a joystick while viewing its real-time replication by a virtual hand, and occasionally manipulated feedback by adding some rotation to the movement^24^. In another study, while perceiving the tactile feedback of one’s own action, it was occasionally delivered by external agents or with some temporal delay^12^. These manipulations typically resulted in activation of relevant sensory areas^20^, such as the bilateral superior temporal gyrus (STG) for auditory feedback^18^, and multimodal or motor-association areas, such as the temporo-parietal junction (TPJ), supplementary motor area (SMA), and inferior frontal gyrus (IFG)^18–21,24–28^. Technically, it is important to note that in these studies, the violated sensory feedback was still contingent on preceding events to a certain degree, namely it was ‘imperfectly contingent.’ Therefore, in the classic contingency-error paradigm, the neural response to contingency error is identified by contrasting the imperfectly contingent condition against the perfectly contingent condition.

Such a neural response to contingency error has so far been explained by two distinct accounts in the framework of the forward model, namely, agency error and prediction error. The two accounts differ in the assumed domain of the error. The agency-error account assumes an error specific to the action agency, so the neural response to contingency error is assumed to reflect the functioning of the entire system for the generation of forward prediction associated with sense of agency. Functioning is assumed to include compensation for action deviation, recalibration of the internal model, or increased self-monitoring^8,9,13–16^. Accordingly, the response is considered to take place primarily in multimodal or motor association cortices^29,30^. In contrast, the prediction-error account assumes an error non-specific to action agency but common across sensory processing, so the neural response to contingency error is assumed to reflect decreased sensory attenuation. This account entails a larger framework of prediction error, in which attenuation of predictable sensory input leads to a behavioral advantage by making unpredictable inputs salient. The unpredictable inputs are meaningful in that they may signal an environmental change and require an appropriate behavioral response (i.e., prey or predator). However, the predictable inputs are meaningless, and their suppression allows the agents to focus their cognitive resources on meaningful inputs^2,20,22,31^. Thus, this account considers contingency error a class of prediction error, and it is primarily interested in the response of unimodal sensory areas.

The two accounts are associated with different views of schizophrenia pathology. The agency-error account is supported by researchers who have attributed functioning of the forward model to self-related pathology^2,16^. In this view, dysfunction exists somewhere in the processes generating forward prediction, ranging from the generation of appropriate motor commands to the calculation of sensory prediction. This view is empirically supported by the reported inability of patients to predict the sensory consequence of an action or compensation for a resulting action deviation^16,32–34^, as well as by the fact that the self-related symptoms of schizophrenia are rarely limited to a single sensory modality^35,36^. In contrast, the prediction-error account emphasizes the abnormal perception of the environment in schizophrenia. A failure to suppress meaningless sensory input may underlie the pathological perceptual experience of ‘heightened awareness’ or ‘apophenia,’ which is assumed to extend to the inability to distinguish between meaningful and meaningless events^37,38^. Consistent with this view, sensory attenuation may be abnormal in the acute phase of schizophrenia^39^ and is associated with dopaminergic functioning in animals^40^. Patients with this symptom may show deficits in electrophysiological responses in the auditory cortex^41^.

To the best of our knowledge, no neuroimaging studies have examined which of the two accounts better explains the neural response to contingency error. Although previous studies have interpreted contingency-error activation using one of the two distinct accounts, all findings have been obtained using a similar classic contingency error paradigm, that is, the comparison of perfectly and imperfectly contingent conditions. Previous studies that have applied the prediction-error account have typically focused their attention on the sensory cortices^20,42^, while those that have used the agency-error account have focused on the multimodal or motor-association cortices^19,24,26^. Theoretically, either only one of the two accounts is correct, or both accounts are correct when applied to different brain regions. This conceptual ambiguity inherent to the classic contingency-error paradigm is a critical challenge that must be overcome to make further scientific and clinical contributions.

In this study, we investigated which account better applies to the neural contingency-error response. To distinguish between the two accounts, we devised new features for the classic contingency-error paradigm in a functional magnetic resonance imaging (fMRI) study using healthy adults. We examined the two neural characteristics that are expected only in the prediction-error account. The first characteristic is the response to an isolated auditory stimulus that is not predictable at all. The response would not occur under the agency-error account because the stimulus is unrelated to the forward model. A larger response to this stimulus than to the imperfectly contingent stimulus is expected by the prediction-error account because the former is more unpredictable. The second characteristic is the response to passive-contingency delay, namely, the delay in the occurrence of an auditory stimulus contingent on a visual cue, rather than the subject’s action. Again, no response is expected under the agency-error account, while a similar level of response to that of the action contingency delay under the prediction-error account is expected because the response is indifferent to the source of prediction^43^. In this study, we first identified cortical regions responsive to contingency error using the classic contingency-error paradigm, and then examined whether these two characteristics existed in each of these regions.

## Results

### Behavioral data

The mean accuracy of the visual response task in our subjects was 98.1% (standard deviation [SD] = 1.93) and the mean reaction time was 437.5 msec (SD = 90.4). The subjects successfully ignored the red square (i.e., correct rejection) in 99.4% (SD = 0.60) of the trials.

### fMRI data

The cortical regions responsive to contingency error (i.e., MI – MP) were identified in the right STG, TPJ, SMA, and bilateral IFG, and the left temporal pole (*p* < 0.001, uncorrected; corrected to *p* < 0.05 using cluster size). Then, to investigate which of the two accounts better applies to the response in these regions, we performed post hoc analyses of the responses to the isolated auditory stimulus and to passive-contingency delay at the peak voxel in each activated region (Table 1, Fig. 2). A larger (*p* < 0.05, uncorrected) response to the isolated auditory stimulus (i.e., N – MI) was observed only in the right STG; all other regions showed significant differences in the opposite direction (i.e., MI – N; note that this test is positively biased). A significant response to passive-contingency delay (i.e., VI – VP) was also observed in the right STG only.

**Table 1:** fMRI results.

Structure		Peak				Cluster size	Post hoc analysis: *T*(*p*)		
		Coordinates (x y z)			*T*	#voxels (*p*)	N – MI	MI – N^†^	VI – VP
Superior temporal gyrus	R	63	−34	4	5.52	297 (<0.001)	5.46 (<0.001)		4.06 (<0.001)
Temporoparietal junction	R	57	−43	22	5.59	*		6.28 (<0.001)	
Supplementary motor area	R	9	14	58	6.50	182 (0.002)		6.05 (<0.001)	
Inferior frontal gyrus	R	45	14	16	6.41	672 (<0.001)		5.37 (<0.001)	
	L	−54	8	19	6.98	91 (0.029)		6.32 (<0.001)	
Temporal pole	L	−48	5	−11	4.96	141 (0.005)		2.07 (0.024)	

For each activation peak of a contingency-error-responsive region, the MNI coordinates (x, y, z), t-value, cluster size (voxel size = 3 × 3 × 5 mm^3^), and its corrected *p*-value are shown. All peaks are from the contrast MI – MP at *p* < 0.001 (uncorrected) and are corrected to a family-wise error of *p* < 0.05 using cluster size. For each peak voxel, the *t*-value (*p*-value) for *t*he *post hoc* analyses, N – MI, MI – N, and VI – VP are given if significant (*p* < 0.05, uncorrected). *Included in the same cluster as the right superior temporal gyrus. ^†^The statistical test was positively biased because the contrast was dependent on the initial sampling contrast (i.e., MI – MP).

**Figure 1:**
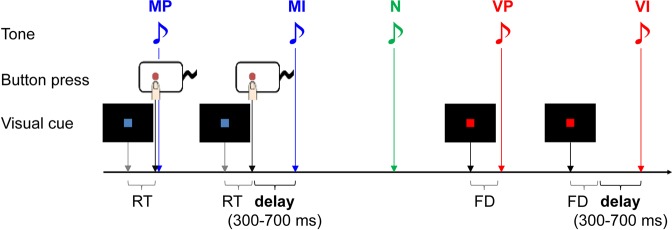
Experimental design. The subjects pressed a button when the blue square (but not the red square) was presented. Five types of pure tones, which appeared to be task-irrelevant to the subjects, were the targets of our analysis. The motor perfect contingent (MP) and motor imperfect contingent (MI) tones were presented contingent on the subjects’ button press; the latter was a rare event that included various (300–700 ms) delays intended to induce the contingency-error response. The non-contingent (N) tone was presented in isolation and was expected to induce a prominent response under the prediction-error account. The visual perfect contingent (VP) and visual imperfect contingent (VI) tones were contingent on the red square. The latter, a rare delayed event compared to the MI tone, was intended to induce a passive-contingency delay response under the prediction-error account. RT, reaction time; FD, fixed delay adjusted to the mean RT in each subject.

**Figure 2:**
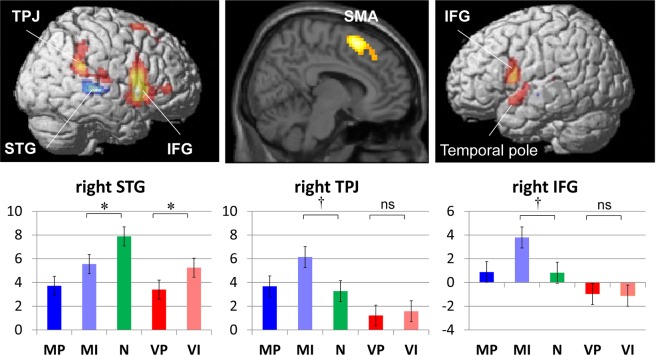
fMRI results. Regions that showed a significant response to contingency error (i.e., MI – MP) were surface-rendered on the right hemisphere, parasagittal section, and left hemisphere (top left, center, and right panels, respectively). Areas that showed a larger response to the isolated auditory stimuli (i.e., N – MI; *p* < 0.05, uncorrected) are shown in blue-white, and those that showed the opposite pattern (i.e., MI – N; *p* < 0.05, uncorrected) are shown in red-yellow. The activation profiles of the right STG, TPJ, and IFG are presented in the bottom left, center, and right panels, respectively. The values are the parameter estimates, and the error bars are the standard error of the mean *p < 0.05, uncorrected. ^†^*p* < 0.05 (uncorrected). The test was positively biased. ns, not significant; STG, superior temporal gyrus; TPJ, temporoparietal junction; IFG, inferior frontal gyrus; SMA, supplementary motor area.

## Discussion

We investigated whether the neural response to contingency error is better explained by the agency-error account or by the prediction-error account. More specifically, we examined whether or not the two neural characteristics expected under the prediction-error account were observed in contingency error-responsive brain regions. Among the regions that exhibited a contingency-error response, responses to isolated auditory stimuli and passive-contingency delay were observed only in the right STG. The rest of the regions, including the right TPJ, right SMA, bilateral IFG, and left temporal pole, did not show either characteristic, suggesting their relevance to the agency-error account. Thus, for the first time, we demonstrated dissociable region-specific accounts of neural responses to contingency error.

The current findings provide further support for the regions as assigned by the agency-error account. For the first time, these regions were explicitly shown to be unrelated to prediction error; that is, the regions were not responsive to simple unpredictability (i.e., N < MI) or visually induced prediction error (i.e., VI ≒ VP). The account primarily assumes the involvement of the multimodal or motor-related areas^16,29,30,44^. Indeed, the regions implicated in this account in the current study (i.e., the right TPJ, SMA, bilateral IFG, and left temporal pole) have been reported to show contingency-error responses across multiple sensory modalities^12,18,26,27,45–49^. Specifically, activation in the bilateral IFG has been implicated in sustained attention as part of the frontoparietal network^26,50^, while activation in the right SMA has been implicated in the planning of movement, motor intentions, and motor preparation^51–53^, and activation in the right TPJ in the detection of action-contingency error^21,24,26,54^.

The current findings provide explicit support for the association of the prediction-error account with the unimodal sensory area. Neural responsiveness to the totally unexpected tone (i.e., N > MI) and visually induced prediction error (i.e., VI > VP) were observed only in the right STG. The right STG region seems to belong to the unimodal auditory cortex, as described in a recent study that investigated the cytoarchitectonic and receptorarchitectonic organization of the human auditory cortex^55^. According to an anatomical toolbox that implements this anatomical dataset^56^ (the SPM Anatomy Toolbox), our right STG peak most likely belonged to the Te3, which is equivalent to the auditory association cortex (Brodmann area 22).

The neural response to contingency error was mostly right-lateralized in this study. This may be explained by the nature of our task as it was likely to involve visuomotor action monitoring and bodily self-awareness, and right-lateralized fronto-parietal activation has been commonly observed in previous studies that involved these processes^21,26^.

Our results provide the first unified picture of the two distinct accounts of the neural response to contingency error. Previous neuroimaging studies that have investigated this issue have adopted either account without making experimental distinctions. The response of the multimodal areas is indeed likely to be specifically relevant to the functioning of the forward-prediction system (i.e., agency-error account), while that in the unimodal sensory areas is more comprehensively explained as the decrease in prediction-induced sensory attenuation (i.e., prediction-error account). The new feature of our experimental design to assess the neural response to isolated sensory stimuli and passive-contingency delay may result in a new standard in this research field.

Our results suggest the utility of the current experimental paradigm in opening a new line of neuroimaging research for schizophrenia. In previous studies, different symptoms were associated with abnormal brain regions using different experimental tasks, but we found that the reported symptom region associations paralleled the account region associations. For example, using the detection task of visuomotor incongruence, patients’ false detections were associated with abnormal activation of the multimodal cortex^23,25^. Using the differentiation task of distorted and undistorted speech, hallucinations were associated with altered activation of the STG^57^. It may be possible to map different symptoms or pathologies using our unified experimental paradigm and associate them with the two accounts for a deeper pathological understanding and diagnosis.

We recognize two major limitations of this study. First, we addressed only auditory contingency error. The cross-modal generalization of our results requires further research investigating other sensory modalities. Second, we used only temporal delay as the violation of contingency. A similar experiment using other types of violations, such as spatial violation, may produce different results. Nevertheless, we consider that the current results are essentially representative because previous studies that have used different modalities of sensory feedback or different contingency-violation methods have provided largely convergent findings^20,24,26,58^.

In conclusion, we demonstrated that the distinct agency-error and prediction-error accounts of the neural contingency-error response apply to different cortical regions. Among the regions that exhibited auditory contingency-error response (the right STG, TPJ, SMA, and bilateral IFG, and the left temporal pole), only the right STG exhibited a response to isolated auditory stimuli and passive-contingency delay, suggesting the relevance of this region to the prediction-error account and other regions to the agency-error account. These findings provide a unified picture of the two accounts and may establish the basis of a new epoch of basic and clinical neuroimaging studies on contingency error.

## Methods

### Subjects

In total, 28 healthy right-handed volunteers with no significant psychiatric or neurological history were studied. They were confirmed as right-handed using the Edinburgh Handedness Inventory. Data from two subjects were excluded due to insufficient task performance (response accuracy < 90%). Thus, data from the remaining 26 subjects (mean age, 21.0 years; range: 18–24 years; 10 females and 16 males) are reported. Informed consent was obtained from all subjects prior to their participation. This study was approved by the Ethics Committee of the Graduate School of Medicine of Tohoku University and was conducted in accordance with the Declaration of Helsinki.

### Task

The task (Fig. 1) was a simple visual response task. The visual stimuli consisted of blue or red squares presented at the center of a screen (200 ms). The subjects had to press a button as quickly as possible when a blue square was presented. There were several task-irrelevant auditory stimuli, which the participants were told were random ‘distractors.’ However, the neural response to the tones was of interest. The auditory stimuli consisted of pure tones (200 ms) with three different pitches (440, 550, and 660 Hz). By manipulating the type of contingent stimulus and delay, five dissociable tone presentations were created. The order and timing of the presentations of the visual cues or tone types were pseudo-randomized. The experimental session consisted of 12 alternations of a 50-s task block and a 12-s rest block, starting with a 3-s rest period. The rest block was indicated by dimming of the central fixation cross. Each subject participated in four sessions, for a total task length of 3042 s.

### Tone types

The tone of the first pitch was contingent on the subjects’ button press. The majority (83.35%) were presented immediately (motor perfect contingent; MP), and the remainder (16.65%) were presented with delays of 300, 500, or 700 ms (equal occurrences) as the deviated condition (motor imperfect contingent; MI). The contrast between MI and MP allowed us to identify neural contingency-error responses according to the classic paradigm.

Next, a tone at a second pitch was presented in isolation with no preceding cue (non-contingent; N). The neural response to this tone was expected to be more prominent than that to the MI tone under the prediction-error account.

Finally, a tone with a third pitch was contingent on presentation of the red square; therefore, it was predicted by the visual cue. The majority (83.35%) occurred after a fixed interval as the standard condition (visual perfect contingent; VP), and the remainder (16.65%) occurred with further delays of 300, 500, or 700 ms (equal occurrences) as the deviated condition (visual imperfect contingent; VI). The contrast between VI and VP was expected to show the effects of passive-contingency delay only under the prediction-error account. The fixed interval preceding the VP or VI tone was adjusted to the mean reaction time of the visual response task in each subject, so that the intervals between the visual cue and the tone in the VP and the MP match, as well as those in the VI and in the MI. This allowed us to attribute differential activation between VI and VP, and between MI and MP, to action contingency alone, rejecting the alternative explanation attributing it to different intervals between visual cues and tones.

Our use of three pitches was intended to ensure the association or non-association of each tone with the preceding cue. The type of preceding events (e.g., visual cues, motor responses, and no preceding events) was coupled with the tone of the specific pitch. In other words, the action was always followed by the tone of the specific pitch (i.e., MP or MI), and the tone had never been presented without the preceding action. We expected that this feature minimized the possibility of participants’ misattributing action-contingency to tones with other types of pitch.

In total, 180 MP, 36 MI, 36N, 180 VP, and 36 VI tones were presented to each subject. The relationship between pitch and tone type was counterbalanced across subjects to avoid the potential effect of the pitch difference on the results. On the day preceding the fMRI experiment, each subject practiced the task using 12 blocks. The practice session had two purposes. The first was to allow participants to sufficiently learn to associate the pitch with the preceding cue types (i.e., own action or visual input). For this purpose, the same pitch and tone type assignment as the fMRI sessions was used. The second purpose was to determine the fixed interval preceding the VP or VI tone in the fMRI experiment. The mean reaction time of the visual response task during the last eight blocks was used as the fixed interval.

### fMRI measurement and image preprocessing

In total, 25 gradient-echo images (echo time = 30 ms, flip angle = 70°, slice thickness = 4 mm, slice gap = 1.00 mm, field of view = 192 mm, and matrix size = 64 × 64) covering the whole brain were acquired with a repetition time of 1500 ms using an echo-planar sequence and a 3 T magnetic resonance scanner (Achieva; Philips Medical Systems, Crawley, UK). During each session, a total of 507 volumes and three initial ‘dummy’ volumes were acquired for stabilization of the T1 effect. A T1-weighted anatomical image was also acquired for each subject.

The following preprocessing procedures were performed using Statistical Parametric Mapping (SPM8) software (Wellcome Department of Imaging Neuroscience, London, UK) implemented in MATLAB R2014a (MathWorks, Natick, MA, USA): correction for head motion, adjustment of acquisition time across slices, coregistration to an anatomical image, spatial normalization using the anatomical image and the MNI template, and smoothing using a Gaussian kernel with full-width at half-maximum of 8 mm.

### fMRI data analysis

All subject-level statistical analyses were performed using a general linear model. Statistical predictors were generated by convolving a series of discrete events with a canonical hemodynamic response function in SPM8. The event was modeled at the onset of the auditory stimulus with no duration for the MP, MI, N, VP, and VI tones. The 50-s task blocks were also included to model sustained attention (relative to the rest period). Head motions were modeled as a covariate. A voxel-by-voxel multiple regression analysis of these predictors was applied to the preprocessed images for each subject. High-pass filtering (cutoff of 128 s) was applied.

For the second-level between-subjects (random effects) model, statistical inference for the contrast of parameter estimates was performed using a one-sample t-test. The neural response to contingency-error was identified by contrasting MI and MP. The statistical threshold was set to *p* < 0.001 (uncorrected) for height and corrected to *p* < 0.05 for multiple comparisons using cluster size.

Once the peak voxels of each contingency-error-responsive region had been identified, we examined the two characteristics that indicated either the agency-error account or the prediction-error account. The response to the isolated stimulus was examined by comparing the response to the N tone with that to the MI tone. The agency-error account predicts a greater response to the MI tone, whereas the prediction-error account predicts a greater response to the N tone. The response to the passive-contingency delay was examined using the contrast between VI and VP. A significant difference in activation was expected only in the prediction-error account. The statistical threshold was set to an uncorrected *p*-value < 0.05.

Activation peaks were labeled mainly based on SPM anatomy toolbox version 2.2b^56^. However, when the labelling was not sufficiently informative, we relied on Automated Anatomical Labelling implemented in SPM8 or visual inspection with reference to the anatomical T1 image.

We did not include separate regressors for the visual cue and participants’ motor responses, because they would have been highly correlated with the regressors for the VI/VP and MI/MP events, respectively, due to the short intervals between the cue and tone. Therefore, the estimated neural activities for the MI, MP, VI, and VP conditions were affected not only by the perception of the tone but also by the perception of the visual cue and the participants’ motor response. Taking this into account, we performed a voxel-wise search only on the MI - MP contrast, in which the effects of the visual perception and motor response were perfectly controlled. Although the contrast of N and MI applied in the post hoc analysis was confounded by the difference in these effects, we considered it not to be problematic because the analysis was conducted in the MI - MP ROIs, which assured that the activation was unrelated to these effects.
